# Protocol for an umbrella review of the state-of-science on public and patient involvement in health and social care research with children, young people and families.

**DOI:** 10.12688/hrbopenres.14142.2

**Published:** 2026-03-23

**Authors:** Shauna Malone Gill, Orla Hayes, Yvonne Corcoran, Anna Gunning, Veronica Swallow, Emma Nicholson, Begonya Nafria Escalera, Amanda Drury, Edel Murphy, Arthur Templeman-Lilley, Veronica Lambert

**Affiliations:** 1 School of Nursing, Psychotherapy and Community Health, Faculty of Science and Health, Dublin City University - Glasnevin Campus, Glasnevin, County Dublin, D09 V209, Ireland; 2 PPI Ignite Network @ DCU, Dublin City University - Glasnevin Campus, Glasnevin, County Dublin, D09 V209, Ireland; 3 Children's Health Ireland (CHI) at Crumlin, Children's Health Ireland, Dublin, Leinster, D12 N512, Ireland; 4 Children in Hospital Ireland, Suite 113 5, 4-5 Burton Hall Rd, Children in Hospital Ireland, Sandyford, Dublin 18, D18 A094, Ireland; 5 Department of Nursing and Midwifery, Sheffield Hallam University, Sheffield, England, S1 1WB, UK; 6 School of Psychology, Dublin City University - Glasnevin Campus, Glasnevin, County Dublin, D09 V209, Ireland; 7 Patient Engagement in Research Department, Institut de Recerca Sant Joan de Déu, Santa Rosa 39-56, 08950, Esplugues de Llobregat, Spain; 8 Public and Patient Involvement (PPI) Ignite Network, Institute for Lifecourse and Society, University of Galway, Galway, County Galway, H91 C7DK, Ireland; 9 Independent Lead Advisor, Wales, UK

**Keywords:** Patient and public involvement, engagement, child, adolescent, family, health and social care research, overview of reviews, review of reviews

## Abstract

**Background:**

Public and Patient Involvement (PPI) refers to the active collaboration of patients and the public in health and social care research decision-making, enhancing research success, cost-effectiveness, and impact. Children, young people, and their families bring unique lived experiences to PPI in research, relating to others with similar experiences, while factors like age, cognitive maturation, and developmental stage create differences between researchers and patients or the public. Collaboration with children, young people, and their families should be guided by specific project context and a strong evidence base. However, existing systematic reviews reveal inconsistencies in reporting and a lack of standardised methods for these groups, limiting effective PPI implementation. This protocol details methods for an umbrella review to identify the current state-of-science and future priorities for collaborating with children, young people and families in health and social care research.

**Methods:**

The protocol was developed in accordance with the Preferred Reporting Items for Systematic Reviews and Meta-Analyses Protocols (PRISMA-P) guidelines, which will also direct the reporting of findings. The Joanna Briggs Institute’s methodology for conducting umbrella reviews will be adhered to throughout. MEDLINE, CINAHL, EMBASE, PsycINFO, Cochrane, DARE, JBI Evidence Synthesis, PROSPERO, grey literature databases, targeted international networks, and the Google search engine will be searched for systematic reviews on PPI with children, young people, and families in health and social care research. Two reviewers will independently conduct eligibility screening, data extraction, and quality appraisal.

**Conclusions:**

This umbrella review will provide critical insights into the state-of-science of PPI with children, young people and families in health and social care research. The synthesis of findings could yield important information for researchers and other stakeholders conducting health and social care research in collaboration with children, young people and families by highlighting key patterns, gaps, and recommendations to guide future PPI practices, policies, and research.

Registration

This umbrella review was registered in PROSPERO, the International Prospective Register of Systematic Reviews (Prospero registration number: CRD42024608935, registered 14th of November 2024).

## Introduction

Health and social care research which aims to improve, repair or maintain the health or lives of a population should be responsive to the requirements and expectations of that population. ^1^ The National Institute for Health Research ( NIHR) defines public and patient involvement (PPI) as *“Public patient involvement is research carried out ‘with’ or ‘by’ members of the public rather than ‘to’, ‘about’ or ‘for’ them”.* PPI treats patients and members of the public as contributing members in the research design rather than merely being research subjects. ^2^ PPI is distinct from research participation and does not involve subjects providing data to test a hypothesis. Its purpose is to involve and engage patients, or members of the public in decision making processes, including design, development, conduct, and/or dissemination of research. PPI has earned international recognition as best practice in research. In order to undertake research, many funders globally specify expectations for incorporating PPI in research in order to gain funding. ^3^

Research continues to build a strong evidence base for PPI, identifying its contribution to better-quality research and enhancing relevance and appropriateness of research. ^4^ PPI ensures that suitable research priorities are established, in addition to improvements in advertising, recruiting, research design, study interpretation, dissemination, outreach and impact. ^5^ Furthermore, it has been documented that the public and/or patients have a right to be involved in the execution, oversight and governance of publicly funded research. ^6^

According to Jones *et al.* precise reporting of PPI supports future research, in addition to improving research standards and best practice. ^7^ Moreover, PPI aids in the reduction of research ‘waste’, ^8^ increases public support and the public’s understanding about methods of effective involvement in research. ^9^

Regarding children and young people, the United Nations Convention on the Rights of the Child ^10^ highlights their right to be heard and listened to on all matters affecting them. This includes having the opportunity to directly influence research through PPI and collaborative activities. This safeguards the relevancy of research thus bringing about results that benefit the research population of interest. Conducting research with children and young people presents distinct challenges which can cause decreased participation and retention, leading to fewer medicine, health care and therapeutic advancements for children and young people. ^11^ Collaborating with children, young people and their families may help address these challenges.

There are a variety of ways in which children and young people may benefit from their collaboration in research, for example, skills acquisition ^12^ ^,^ ^13^ or gaining confidence. ^14^ These examples support children and young people in exercising their rights under the United Nations Convention on the Rights of the Child, particularly Article 12 - the right to be heard and Article 29 - the right to develop their personality, talents, and abilities to their fullest potential. Additionally, children and young people gain an opportunity to impact guidelines and clinical practice thus benefiting them in an indirect, but meaningful way. ^13^ The primary challenge when collaborating with children and young people is to ensure their involvement is authentic and not a tick box drill. ^15^ By applying the four principles of the Lundy Model - Space, Voice, Audience, Influence - researchers can create meaningful opportunities that move beyond tokenism and empower children and young people. ^16^

System-wide implementation of collaborating with children and young people and their families should be guided by high-quality evidence. Recent systematic reviews on the engagement of children and young people in research have highlighted that their involvement leads to more relevant and impactful research, enhanced healthcare services, and improved health outcomes for children and young people. ^17^ ^,^ ^18^ However, reviews also highlight a significant gap in standardized methods and reporting practices related to involving children and young people in research decision-making processes. ^17^ ^–^ ^20^ It remains unclear as to if and how PPI activities are reported. This makes it difficult for both researchers and clinicians to critically appraise existing PPI research involving children and young people. Additionally, this lack of standardized reporting complicates the ability of researchers and clinicians to integrate meaningful PPI reporting into their work. ^20^ As a result, there is a pressing need for a summary of how PPI methodologies in research collaborating with children and young people are reported so structured frameworks that can guide the inclusion of children and young people in decision-making, ensuring transparency and consistency in reporting practices can be further developed and utilized. Addressing this gap will ultimately improve the quality of research and its applicability in clinical settings, enhancing the role of children and young people in shaping decisions that affect their health and well-being. ^20^

This paper details a protocol for an umbrella review of systematic reviews which will provide an expansive and comprehensive overview of the current state-of-the-science and future priorities for public and patient involvement in health research with children, young people and families. Collaborating with children, young people and families spans diverse contexts, including healthcare, education, and community services. By examining existing systematic reviews, this umbrella review can integrate findings across these areas. Therefore, this review will serve as a valuable resource for synthesizing a large body of research, summarising methodologies of involvement, identifying patterns and inconsistencies in the evidence, and providing actionable recommendations. This comprehensive review would fill a critical gap in the literature and help shape future practices, policies, and research aimed at enhancing the involvement of children and young people in health and social care research.

## Protocol

We developed and reported this umbrella review protocol in accordance with the Preferred Reporting Items for Systematic review and Meta-Analysis Protocols (PRISMA-P). ^21^ The PRISMA-P checklist associated with this protocol is available on the Open Science Framework (OSF) under the project title: “PRISMA-P Checklist Protocol for an umbrella review of the state-of-science on public and patient involvement in health and social care research with children, young people and families” available at: https://doi.org/10.17605/OSF.IO/ZVWES. ^22^

This umbrella review was registered in PROSPERO, the International Prospective Register of Systematic Reviews (Prospero registration number: CRD42024608935, registered 14th of November 2024).

We will follow the Joanna Briggs Institute (JBI) methodology of umbrella reviews guidance, ^23^ including the following procedural steps: eligibility criteria, search strategy, study screening and selection, quality appraisal using a suitable tool, data abstraction using a tabulated matrix and synthesis and reporting of results and discussion. An umbrella review is an overview of existing systematic reviews, with JBI Umbrella Reviews designed to incorporate all types of syntheses of research evidence, including systematic reviews in their various forms. The finding of this review of reviews will be informed and reported according to the Preferred Reporting Items for Systematic Reviews and Meta-Analyses (PRISMA) 2020 statement: an updated guideline for reporting systematic reviews. ^24^

### Eligibility criteria

Identified reviews will be included based on the following eligibility criteria:

***Population.*** The review study population will include children and young people up to, and including, the age of 24 years (drawing on the United Nations Convention of the Rights of Children (1989) definition of child as under 18 years and the World Health Organisation definition of young people as between 10 and 24 years). Although the authors recognise that there are a number of different family arrangements including two parent/guardian households, single-parent/guardian families and blended families (where children may have different parents/multi-generational households). This umbrella review will include reviews that include parents/guardians and/or immediate family members (i.e., siblings) of children and young people aged 0–24 years. In relation to the age-cut off for children and young people, it is possible that only a subset of primary studies within the included systematic reviews will meet the Umbrella Review’s eligibility criteria (i.e., where the scope of the Umbrella review is narrower than the scope of one or more of the relevant systematic reviews). Therefore, studies that include adults will be considered if the primary focus and majority of participants pertain to individuals aged 0–24 years. While it might not always be possible to foresee when this situation might occur we will consider each relevant systematic review on a case by case basis and document any post hoc decisions in our Umbrella Review.

***Phenomena of interest.*** The phenomenon of interest is PPI which is where the public and patients are actively and meaningfully involved in decision-making as equal partners across all stages of the research process. For example, PPI is about people actively contributing through discussion to decisions about research design, acceptability, relevance, conduct and governance from study conception to dissemination. ^25^ Within the European Commission’s framework, the Clinical Trial Regulation encourages patient involvement and incorporates this into the assessment of the clinical trial application. According to Regulation 536/2014, Annex 1, Article 17(e), a description of patient involvement in the clinical trial design is required. ^26^ For the purpose of this review, we will draw on the UK National Institute of Health Research Centre for Engagement and Dissemination (NIHR CED), formerly INVOLVE, to define PPI as research being carried out ‘with’ or ‘by’ children, young people and their family members rather than ‘to’, ‘about’ or ‘for’ them. PPI is seen as an active partnership between researchers and patients, carers, and public members (in this case children, young people and family members of children and young people) that shapes and influences the research. The term involvement is often used interchangeably internationally with words such as engagement and participation. We will exclude reviews that focus on the recruitment of children, young people and family members of children and young people as participants in research and/or where information, knowledge exchange or dialogue about research is communicated and disseminated with public communities (e.g., science festivals, raising awareness of research through media).

***Context.*** We will consider health and social care research in any setting (acute, primary health care, medical paediatric specialties, community, etc.). Health research is defined as research undertaken to acquire knowledge of health and disease, spanning biological mechanisms, population health, disease prevention, diagnosis, treatment and care of children, young people and their family members, including health services. ^27^ Social care research is defined as any research relating to personal care or other practical assistance because of age, physical or mental illness, disability, pregnancy, childbirth, dependence on alcohol or drugs or other similar circumstances. ^28^

***Outcomes.*** Drawing on the second Guidance for Reporting Involvement of Patients and the Public (GRIPP2) long form for reporting public and patient involvement in research, ^29^ outcomes will include types of PPI methods/approaches; level of PPI involvement (e.g., consult, collaborate, co-lead); stage of involvement in the research process; impacts (positive and negative) of PPI on the research, individuals (i.e., PPI contributors, researchers) involved and wider impacts, including how these are measured; barriers and facilitators to, and types of training and support mechanisms for, integrating PPI with children, young people and their family members.

***Study design.*** Systematic reviews, and comprehensive reviews of any design type (e.g., scoping reviews, rapid reviews, integrative reviews etc.) with evidence of a systematic search strategy in at least two databases, eligibility criteria for inclusion and exclusion and published in peer-reviewed journals only will be included in the review. Narrative review articles that do not follow a systematic approach, and primary studies will be excluded.

### Patient and Public Involvement (PPI) strategy

We will actively engage children, young people, and their families in the umbrella review process, guided by Independent Lead Advisor Arthur Templeman-Lilley. A collaborative approach will ensure meaningful involvement across various stages, including research prioritisation, data collection, analysis, interpretation, and dissemination of findings. Engagement will follow the guidance provided in the ‘Ethical Collaboration with Children and Young People in Research’ framework. ^30^ Our PPI approach will be informed by a recognised framework (e.g., the Involvement Matrix ^31^) to clearly map and report the level of collaboration.

PPI recruitment will purposefully target a diverse range of contributors, including those from minority ethnic backgrounds and children and young people with disabilities, to ensure a breadth of lived experience. Panel membership will reflect the review’s age range (0–24 years) by including children, young people, and parents/carers, recruited primarily on the basis of lived experience of health and social care services.

Once a PPI panel is recruited, an initial meeting will introduce panel members to the protocol, providing options to ensure the review reflects their perspectives. Training will be offered to enhance research literacy, including skills in summarising findings. Throughout the review, panel members will provide continuous oversight and communications. Additionally, they will contribute to writing child- and young person-friendly summaries of both the protocol and final review findings, ensuring accessibility and impact.

Regular feedback and recognition will be provided throughout the review process to support meaningful involvement. Recognition of PPI contributions will follow established authorship guidance. Our Youth Lead Advisor is formally listed as a co-author for the protocol and future review, and additional PPI contributors who meet authorship criteria will be offered co-authorship. Contributors who do not meet these criteria will be formally acknowledged for their specific roles in both the manuscript, child-friendly summaries and all other mediums of dissemination.

### Search strategy

We will search the following citation databases MEDLINE, CINAHL, EMBASE, PsycINFO, and systematic review repositories such as the Cochrane Database of Systematic Reviews, the Database of Abstracts and Review Effects (DARE), the PROSPERO register and the JBI Evidence Synthesis. We will tailor the search strategies for each database using a combination of free text words and controlled vocabulary (e.g., MeSH) terms representing the following four search components: (i) population group (e.g., child, young person, parent, family); (ii) phenomena of interest - public and patient involvement (e.g., involvement, engagement, participation etc.); (iii) the context/field of focus (e.g., health and social care research); and (iv) review type. The Boolean operator “OR” will be used to combine the selected keywords within each of the search components, and all four concepts will be combined using “AND”. The search will be limited by publication date within the past eleven years (i.e., from 2014 to 2024) to assess the current state of science, to peer-reviewed journals only and to English language, This is a limitation of the review that may introduce language bias and under-represent evidence from non-Anglophone settings, but is necessary due to resource constraints for translation. The search strategy will initially be developed for the Medline database and once tested will be adapted for application to the other databases. An example search strategy for MEDLINE, developed with a librarian, is shown in Additional File 2. Additional review articles will also be sought by screening the reference lists of included reviews for relevant citations.

Guidance for a JBI Umbrella Review states that it should include a search of at least two or three relevant sources for grey literature reports. ^32^ Consequently, we will search the following sources for grey literature: (i) grey literature databases (e.g., Open Grey); (ii) targeted international networks (e.g., INVOLVE/NIHR, Generation R, Wellcome); and (iii) Google search engine (limited to the first 10 pages of hits, representing 100 results, to capture the most relevant hits as well as being a feasible amount to screen).

### Study screening and selection

We will import search outputs into Covidence where a two-part screening process will be undertaken once duplicates have been removed. Part one screening will include two reviewers independently screening review titles and abstracts using the eligibility criteria for this review outlined above. Any discrepancies between reviewers will be resolved by consensus or through discussion with a third reviewer acting as arbitrator. If no abstract is available, we will source and assess the full-text article. For articles deemed to meet the inclusion criteria, full texts will be obtained. For part two screening, two reviewers will independently assess full text review articles against the eligibility criteria before a final decision regarding inclusion or exclusion is confirmed. Any discrepancies will be resolved by consensus or through discussion or with a third reviewer acting as arbitrator as required. We will record reasons for excluding articles at the full-text stage. An adapted PRISMA flow chart will be used to report the screening and selection process at each stage of the review. Decisions regarding the inclusion of reviews with mixed adult and child/young person populations will be documented in a review decision log, detailing the justification for inclusion or exclusion and the reviewers involved.

### Quality appraisal

We will assess the methodological quality of the included reviews using the validated measurement tool for the ‘Assessment of Multiple Systematic Reviews’ (AMSTAR). ^33^ AMSTAR is an 11-item questionnaire which assesses the methodological rigour of each review according to a number of factors including ‘a priori’ design, duplicate study selection and data extraction, comprehensive literature search, inclusion criterion, list and characteristics of studies included and excluded, scientific assessment of included studies, appropriate synthesis methods, publication bias and conflict of interest. We will assess each item against a rating of yes, no, can’t answer or not applicable to identify whether that item is addressed within the review paper. Two reviewers will independently conduct the quality appraisal of the included reviews. Any disagreements will be resolved by consensus or through discussion with a third reviewer acting as arbitrator as necessary. We will not exclude review articles on the basis of their quality, rather the quality appraisal will assist us in judging the strength of evidence generated by the included reviews.

### Data extraction

Information will be extracted from each review using tabulated matrixes, adapted from the JBI exemplar data extraction template, which will be piloted, to include characteristics of review methods and review findings. For characteristics of review methods this will include: author/year/country, review typology, PPI focus/aim of the review, databases searched/search period, number of included studies, number of participants and study designs of included studies. Data abstraction specific to our phenomenon of interest, population details and outcomes will include:

• PPI methods/approaches used to involve children, young people and their family members
• PPI contributors (children, young people and family members) involved and how they were recruited
• At what stage, and how PPI was used throughout the research process. Stage of involvement will be described using the following categories: identifying & prioritising; designing & managing; funding & commissioning; undertaking & analysing; dissemination & knowledge translation; implementing and evaluating impact. ^34^
• Level of involvement of children, young people and family members and how they are involved in contributing to the research (e.g. inform, consult, involve, partnership). ^35^
• Outcomes and impacts (positive and negative) of PPI on the research, individuals (i.e., PPI contributors, researchers) involved and wider impacts, including influence of PPI on research processes, results and outcomes, broader impacts, and any economic or personal effects, along with how these impacts are measured.
• Factors enabling or hindering PPI with children, young people and their family members.
• Training and support mechanisms to facilitate the integration of PPI with children, young people and their family members.

Any additions or modifications to the data extraction tool will be reviewed by all reviewers and discussed in detail before extracting data independently. Two researchers will independently extract data which will be mapped in tabulated matrixes with any discrepancies resolved through discussion until consensus is reached, with a third reviewer acting as arbitrator if required. In line with JBI umbrella review guidance extraction and presentation of data will be limited to the results and findings presented by the included review syntheses (i.e., we will not retrieve primary studies that were included in the included reviews).

### Data synthesis/summary

We will use a narrative analysis and summary method for this umbrella review. A meta-analysis will not be performed as the aim of an umbrella review is to present a summary of existing research syntheses and not to conduct any further meta-analyses of results. ^30^ We will present the findings in tabular format to summarise the key characteristics from each included review and the findings from the reviews will be mapped to the outcomes identified in this umbrella review. As recommended in the JBI guidance for umbrella reviews, where quantitative data are reported we will present tabular data of overall effect estimates or other similar numerical data alongside reporting the number of studies that inform the outcome, the number of participants from included studies and the heterogeneity of the results of included reviews as relevant. For qualitative data, we will present the final and overall synthesised findings from included reviews in tabular format and provide detailed contextual information alongside each synthesised finding. We will also provide a clear indication of any overlaps of original research studies in each of the included research syntheses by conducting a comparison of included studies for each review using a citation matrix. The extent and nature of this overlap will be described narratively and critically considered during the synthesis process. The narrative summary aims to examine and integrate findings from multiple different reviews in order to provide an overview of the current state of science on PPI with children, young people and their family members in health and social care research.

## Discussion

We will summarise the evidence from the included reviews on public and patient involvement in health and social care research with children, young people and their family members. Reporting of findings will include a detailed description of the included reviews, along with an assessment of their methodological quality and an overview of the review characteristics and outcomes. Findings will be discussed in the context of current literature. Similarities and differences across included reviews for PPI methods/approaches, PPI contributor involvement, stage and level of involvement, outcomes and impacts of involvement, enabling and hindering factors to involvement and training and support mechanisms for involvement will be discussed. Implications for PPI best practices, education, policy and research will be presented. Any limitations of the reviews included in the umbrella review, and of the umbrella review itself, will be outlined. Where relevant, differences in methodological processes and quality assessment of included reviews will be discussed, and implications drawn. The review will specifically identify and extract data regarding the inclusion of marginalised or underrepresented populations. A dedicated reflection on these evidence gaps will be included in the final manuscript. We anticipate the findings will be used to inform priorities and recommendations for future research and best practices internationally for PPI in health and social care research with children, young people and family members of children and young people.

## Declarations

### Ethics approval and consent to participate

Ethical approval and consent were not required.

## Abbreviations

AMSTAR: Assessment of Multiple Systematic Reviews; JBI: Joanna Briggs Institute; PPI: Public and Patient Involvement; PRISMA-P: Preferred Reporting Items for Systematic Review and Meta-Analysis Protocols; PROSPERO: International prospective register of systematic reviews; GRIPP: Guidance for Reporting Involvement of Patients and the Public

## Underlying data

No data associated with this article.

## Reporting guidelines

PRISMA-P Checklist associated with this article is available on OSF, under the project title “PRISMA-P Checklist Protocol for an umbrella review of the state-of-science on public and patient involvement in health and social care research with children, young people and families” available at: https://doi.org/10.17605/OSF.IO/ZVWES.

Data are available under the terms of the Creative Commons Zero “No rights reserved” data waiver (CC0 1.0 Public domain dedication).
