# Supplementary material for: Protocol for an umbrella review of the state-of-science on public and patient involvement in health and social care research with children, young people and families
Source: HRB Open Res. 2026 Mar 23;8:78. Originally published 2025 Jul 14. [Version 2] doi: 10.12688/hrbopenres.14142.2 (PMC12405837; doi:10.12688/hrbopenres.14142.2)
Supplement: Peer review history

# Reviewer report: Reviewer response for version 2

**DOI:** 10.21956/hrbopenres.15840.r54280

**Published:** 2026-03-31

**Authors:** Marina Charalambous

**Affiliations:** 1 Rehabilitation Sciences, Cyprus University of Technology, Limassol, Cyprus

The authors have addressed all my comments and I have no further remarks. Good luck with indexing.

Is the study design appropriate for the research question?

Partly

Is the rationale for, and objectives of, the study clearly described?

Yes

Are sufficient details of the methods provided to allow replication by others?

Partly

Are the datasets clearly presented in a useable and accessible format?

Yes

Reviewer Expertise:

Patient and Public Involvement (PPI) in People with Aquired Communication Disorders, PPI Framework Developement and Implementation

I confirm that I have read this submission and believe that I have an appropriate level of expertise to confirm that it is of an acceptable scientific standard.

# Reviewer report: Reviewer response for version 1

**DOI:** 10.21956/hrbopenres.15543.r49128

**Published:** 2025-09-02

**Authors:** Cuma Çakmak

**Affiliations:** 1 Dicle University, Diyarbakır, Turkey

Public and patient participation is an important issue. In fact, more research is needed in this area. While there has been an increase in studies such as patient participation, the number of such new studies needs to increase. This is a robust and well-designed protocol with the potential to inform future research and practice on PPI with children and their families. 

 The study has been prepared with considerable strengths. However, this situation can be overcome with some simple corrections. It is also beneficial to mention these as limitations of the study.

 First of all, the choice of only English may be a limitation.

 A flowchart also could be created (for eligibility section)

 There are some punctuation errors in the article, which can be corrected.

Is the study design appropriate for the research question?

Yes

Is the rationale for, and objectives of, the study clearly described?

Yes

Are sufficient details of the methods provided to allow replication by others?

Yes

Are the datasets clearly presented in a useable and accessible format?

Yes

Reviewer Expertise:

Patient engagement, health management

I confirm that I have read this submission and believe that I have an appropriate level of expertise to confirm that it is of an acceptable scientific standard.

## References

[1] : Protocol for an umbrella review of the state-of-science on public and patient involvement in health and social care research with children, young people and families. *HRB Open Research* .2025;8: 10.12688/hrbopenres.14142.1 10.12688/hrbopenres.14142.1 40910072 PMC12405837

# Reviewer report: Reviewer response for version 1

**DOI:** 10.21956/hrbopenres.15543.r48910

**Published:** 2025-09-01

**Authors:** Jana Bergholtz

**Affiliations:** 1 University of Gothenburg Centre for Person-Centred Care, Gothenburg, Sweden

This protocol provides a detailed plan for an umbrella review of systematic reviews on patient and public involvement (PPI) in health and social care research with children, young people, and their families to identify current state-of-science and future priorities. The authors argue that while PPI is increasingly recognized, evidence on *how* children, young people and families are engaged, at what stages, and with what impacts remains fragmented and inconsistently reported. The review aims to provide a comprehensive synthesis of PPI methods/approaches, recruitment, stage of involvement in the research process, levels of involvement, impacts (positive and negative; of PPI on the research, individuals involved and wider impacts, including how these are measured), barriers and facilitators, and types of training and support mechanisms. The protocol adheres to appropriate guidance, is registered with PROSPERO, and integrates a strategy to involve children, young people and families throughout the review process.

 The topic is highly important and timely, but I would like to raise a few concerns:

 1. Angle of the review considering recently published umbrella reviews

 As it stands, this umbrella review risks partly duplicating existing umbrella reviews (1,2 - Warraitch et al. 2024a,b – not cited in the protocol; Wyatt et al. 2024 – cited in the protocol) that already synthesized methods/stages, positive impacts, and challenges of youth involvement in health research. Please reflect on how you could add value with a new umbrella review. A suggestion would be to create lay language summaries/presentations of the existing umbrella reviews and discuss with your youth/PPI panel: Which research questions would they like to see addressed?

 Would they like to see more research on negative impacts (which are mentioned in the protocol but maybe you could elaborate?). *Are* negative impacts being published? If so, are there any negative impacts on individuals involved, e.g. for PPI contributors if involvement feels tokenistic or for researchers if involvement activities lead to ethical stress? This could lead to further discussion questions with your PPI panel, such as: How can negative impacts be prevented? Is GRIPP2 helpful for reporting? How could GRIPP2 reporting guidance be improved and adjusted for children and youth?

 2. PPI strategy

 Please give details on the level of involvement (for the independent lead advisor and/vs PPI panel) throughout the research process (for example a good tool to use is the involvement matrix https://www.kcrutrecht.nl/involvement-matrix/).

 How exactly will you involve children? Which ages? Will it be required for PPI contributors joining the PPI panel to have own personal experience of being involved in health research? (so that they have a deeper understanding what the review is about) Also please reflect on: How can you make sure to keep PPI contributors engaged throughout the research process as they may have many other competing demands and interests. Could this potentially be a school project for them?

 3. Data extraction

 Level of involvement: please clarify if patient-/youth-led research would be included or excluded.

Is the study design appropriate for the research question?

Yes

Is the rationale for, and objectives of, the study clearly described?

Partly

Are sufficient details of the methods provided to allow replication by others?

Yes

Are the datasets clearly presented in a useable and accessible format?

Not applicable

Reviewer Expertise:

patient and public involvement (PPI in healthcare and PPI in research, both mostly from a patient perspective); person-centred care

I confirm that I have read this submission and believe that I have an appropriate level of expertise to confirm that it is of an acceptable scientific standard, however I have significant reservations, as outlined above.

## References

[1] : An umbrella review of reviews on challenges to meaningful adolescent involvement in health research. *Health Expectations* .2024;27(1) : 10.1111/hex.13980 10.1111/hex.13980 PMC1082174339102665

[2] : Positive Impacts of Adolescent Involvement in Health Research: An Umbrella Review. *Journal of Adolescent Health* .2024;75(2) : 10.1016/j.jadohealth.2024.02.029 218-230 10.1016/j.jadohealth.2024.02.029 38597838

# Reviewer report: Reviewer response for version 1

**DOI:** 10.21956/hrbopenres.15543.r48609

**Published:** 2025-08-26

**Authors:** Marina Charalambous

**Affiliations:** 1 Rehabilitation Sciences, Cyprus University of Technology, Limassol, Cyprus

This protocol addresses a highly relevant and timely topic - the involvement of children, young people (CYP), and their families in health and social care research through public and patient involvement (PPI). The protocol is methodologically rigorous, clearly written, and adheres to recognized standards (PRISMA-P, JBI, AMSTAR), demonstrating a strong commitment to co-production and inclusive research practice. It has strong potential to make a valuable contribution to PPI methodology and guidance in paediatric and family-oriented research contexts.

 I commend the authors on the quality and clarity of the protocol. I have identified a few areas where further clarification or minor additions could enhance transparency, reproducibility, and inclusiveness.

 Major Strengths

- The topic is highly significant and addresses an existing gap in the literature on structured and meaningful involvement of CYP and families in research.
- The protocol is grounded in recognised standards (PRISMA-P, JBI methodology for umbrella reviews, AMSTAR quality appraisal), ensuring rigour.
- The planned involvement of a PPI panel, including CYP and families, across various review stages is exemplary and reflects contemporary standards in participatory research.
- The population, phenomenon of interest, context, outcomes, and study design parameters are articulated and justified.
- The use of multiple databases and grey literature sources ensures a thorough and systematic approach.
- The inclusion of data points such as levels and stages of involvement, recruitment methods, impacts, and barriers/facilitators shows thorough planning for a comprehensive synthesis.

Areas for Improvement and Clarification

- The restriction to English-language publications is understandable due to resource but it may introduce bias. A brief acknowledgement of this limitation and its implications for findings (e.g., under-representation of non-Anglophone settings) in the discussion section would strengthen transparency.
- While the authors mention that they will compare included studies across reviews, it would be helpful to specify how they plan to manage overlap (e.g., using a citation matrix or corrected covered area calculation) to avoid duplication bias in the synthesis.
- The protocol describes the meaningful and planned involvement of CYP and their families in the review process. However, it is unclear if they were involved in the development of the protocol itself. If not, acknowledging this and outlining the rationale would be transparent and aligned with PPI best practice.
- The inclusion of child- and young-person-friendly summaries is commendable. The authors may consider briefly outlining dissemination channels (e.g., schools, youth advisory groups, clinical networks) and the possibility of co-authorship or formal acknowledgement for PPI contributors.
- Although formal ethics approval is not required, a short comment on how ethical issues related to involving CYP (e.g., consent, safeguarding, appropriate recognition) will be managed could strengthen the PPI section.
- The approach to reviews that include both adults and children is described as case-by-case. Further detail on how these decisions will be documented and justified (e.g., via a review log) would enhance rigour.
- A brief reflection on how the review process and PPI involvement will consider inclusion of marginalised or underrepresented CYP (e.g., those with disabilities, from minority ethnic backgrounds) would strengthen the protocol’s inclusivity.

This is a methodologically sound, ethically grounded, and well-articulated protocol. With minor clarifications as noted above, it will be well-positioned to deliver a comprehensive and impactful synthesis of the evidence on PPI with children, young people, and families.

Is the study design appropriate for the research question?

Partly

Is the rationale for, and objectives of, the study clearly described?

Yes

Are sufficient details of the methods provided to allow replication by others?

Partly

Are the datasets clearly presented in a useable and accessible format?

Yes

Reviewer Expertise:

Patient and Public Involvement (PPI) in People with Aquired Communication Disorders, PPI Framework Developement and Implementation

I confirm that I have read this submission and believe that I have an appropriate level of expertise to confirm that it is of an acceptable scientific standard, however I have significant reservations, as outlined above.
